# Atypical monoarthritis presentation in children with oligoarticular juvenile idiopathic arthritis: a case series

**DOI:** 10.1186/s12969-016-0129-x

**Published:** 2017-01-13

**Authors:** Natasha Lepore, Megan Cashin, Debra Bartley, Daniela Simona Ardelean

**Affiliations:** 1 Department of Pediatrics, University of Western Ontario, London, ON Canada; 2 Lawson Health Research Institute, London, ON Canada; 3 University College Cork, Cork, Ireland; 4 Western Bone & Joint Institute, London, ON Canada; 5 Department of Surgery, Division of Orthopedic Surgery, London, ON Canada; 6 Department of Medical Biophysics, University of Western Ontario, London, ON Canada

**Keywords:** Children, Atypical monoarthritis, oligoJIA, Presentation, Hip, Elbow, Wrist

## Abstract

**Background:**

Oligoarticular juvenile idiopathic arthritis (oligoJIA), the most common chronic inflammatory arthritis of childhood, usually involves the knees and ankles. Severe oligoJIA monoarthritis presenting in a joint other than knees and ankles, is rare.

**Findings:**

We report four children who presented with severe isolated arthritis of the hip, wrist or elbow and were diagnosed with oligoJIA. All four were girls with a median age of 11.5 years. Those with hip arthritis also met the classification criteria for juvenile-onset spondylarthopathy. Median duration of symptoms prior to diagnosis was 9.5 months. Three children had already cartilage loss or erosive disease at diagnosis.

**Conclusions:**

Children diagnosed with oligoJIA that present with monoarthritis of the hip, wrist and elbow can have aggressive disease. Girls with positive HLA-B27 presenting with isolated hip arthritis could meet the classification criteria for both oligoJIA and juvenile-onset SpA. Early referral to specialized care may improve their diagnosis, treatment and outcome.

## Introduction

Juvenile idiopathic arthritis (JIA) is a heterogeneous group of seven immune-mediated chronic inflammatory diseases that target the joints in children younger than 16 years of age [1]. In Canada and worldwide, the estimated prevalence of JIA is approximately 1 per 1000 children [2, 3].

Almost half of JIA patients are diagnosed with oligoarticular juvenile idiopathic arthritis (oligoJIA) [4]. OligoJIA affects one to four joints within the first 6 months of disease [1]. The common presentation is monoarthritis of the knee in girls younger than 6 year-old, often in association with positive anti-nuclear antibodies (ANA). OligoJIA presenting with erosive monoarthritis in joints other than knees and ankles is a rare event. Very few reports of such cases have been published to date. However, these cases may have poor prognosis [5]. Hence, early recognition is paramount in avoiding irreversible joint and bone damage. In this study, we describe four children with oligoJIA who presented at our center with severe, erosive monoarthritis.

## Findings

Among 53 children with JIA included in an inception cohort established in Sept. 2015, 25 (47%) were diagnosed with oligoJIA. Four of these 25 children (16%) presented with monoarthritis of the hip, wrist or elbow. All were girls. Median age at diagnosis was 11.5 ± 3.5 years (range 9–16 years). Median duration of symptoms prior to diagnosis was 9.5 ± 14 months. Median follow-up was 5.5 ± 3.5 months. All four patients had normal blood cell counts, inflammatory markers, immunoglobulins and rheumatoid factor (RF). Two children had anti-nuclear antibody (ANA) positive at 1:160. Two had human leukocyte antigen HLA-B27 positive and hip arthritis. None had uveitis. Three out of four (75%) children had second degree relatives with rheumatologic diseases. The presenting features of these four children are summarized in Table 1.

**Table 1:** Characteristics at presentation of children with atypical isolated oligoarticular juvenile idiopathic arthritis

Age at dx (ys) & gender	Joint	FU (ms)	Duration of symptoms prior to dx (ms)	Serology	Imaging at presentation		Treatment
					X-ray	MRI	
14, F	L hip	10	36	ANA-; RF -; B27+	Shortening of femoral neck, premature physis closure	Synovitis, ↓ joint space, ↓ cartilage	Naproxen, IAS x 2, MTX, Etanercept
9, F	L hip	5	5	ANA+; RF-; B27+	Normal	Synovitis, subchondral femoral head changes	Indomethacin
16, F	R wrist	10	8	ANA-; RF- B27*	Normal	Synovitis, bone cysts, erosion	Naproxen, IAS x 3
9, F	L elbow	5	11	ANA+; RF- B27*	↓ joint space, atrophy of the elbow complex	Synovitis, ballooning of the humeral epiphysis, multiple erosions	Naproxen, IAS

*ANA* antinuclear antibody, *B27* HLA-B27, B27*: HLA-B27 not done, *dx* diagnosis, *F* female, *FU* follow-up, *IAS* intra-articular steroid injection, *L* left, *ms* months, *MRI* magnetic resonance imaging, *R* right, *RF* rheumatoid factor, *ys* years

### Case 1

A 14 year-old girl has had referred left knee pain for 3 years. In the last year, she developed left groin pain and limping that responded partially to ibuprofen. She had no history of sacroiliac (SI) joint tenderness, inflammatory lumbosacral pain, or symptomatic anterior uveitis. At presentation, Trendelenburg and log roll tests were positive on the affected side and the movements of the left hip were significantly reduced. There was no enthesitis. HLA-B27 was positive, ANA and RF were negative. PPD testing was negative.

**Fig. 1:**
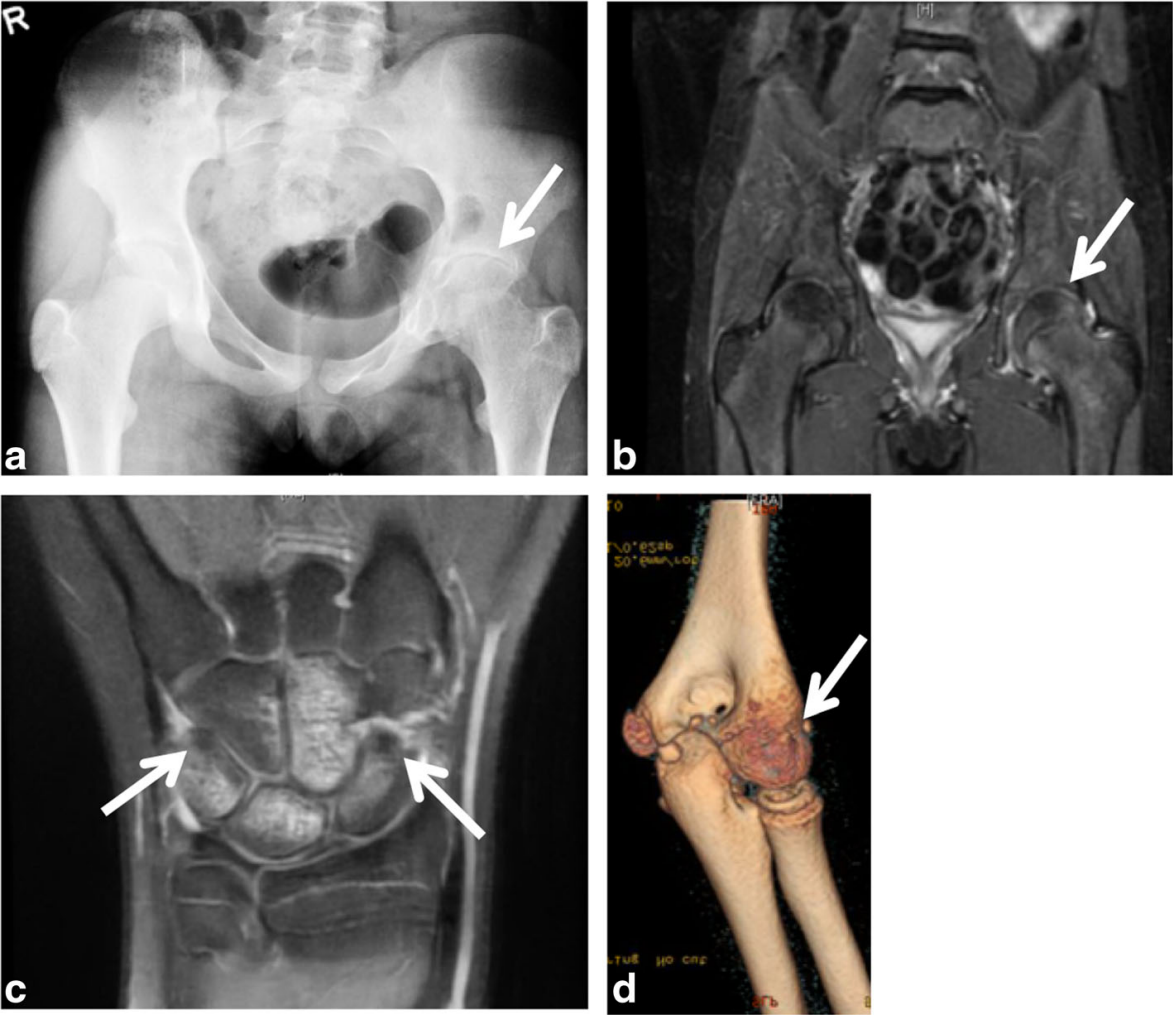
Representative images of severe monoarthritis in children diagnosed with oligoarticular juvenile idiopathic arthritis. **a** X-ray of the hips showed shortening of the left femoral neck and decreased joint space; **b** MRI with gadolinium revealed enhancement and mild thickening of the left hip synovium on T2-weighted imaging; **c** MRI of the right wrist demonstrated carpal synovitis, marked bone marrow edema, bone cysts and erosions on T1-weighted imaging; **d** Non-contrast CT of the left elbow showed bone erosions, hyperostosis of the trochlear-olecranon complex and reduced joint space. *White arrows* point towards the abnormal findings

X-ray of the pelvis done 6 months prior to presentation showed shortening of the left femoral neck, premature closure of the proximal femoral physis and decreased hip joint space (see Fig. 1a). MRI with gadolinium at presentation demonstrated diffuse synovial enhancement, marked joint space loss and subchondral edema of the femoral head and acetabulum. The SI joints were normal. In addition to naproxen, the child required two intra-articular triamcinolone hexacetonide injections and 25 mg of weekly subcutaneous methotrexate to control her hip inflammation. Repeat MRI showed significant improvement in the bone marrow edema of the femoral head and acetabulum, but not change in synovial thickening and enhancement. There was no sacroiliitis. As she continued to have left hip pain with movements, etanercept was added to her regimen.

### Case 2

A 9 year-old girl has had 5 months history of left groin pain with activity. She had no history of SI joint tenderness, inflammatory lumbosacral pain, symptomatic anterior uveitis, or systemic features. On examination, the child had a slight limp and reduced movements of the left hip. There was no enthesitis. HLA-B27 was positive, ANA titers were 1: 160, and RF was negative. The x-ray of the left hip was normal. MRI of the pelvis showed left hip synovitis, trace amount of fluid and subchondral changes within the femoral head (see Fig. 1b). The SI joints were normal. She was started on indometacin with good results. A repeat MRI with gadolinium of the pelvis is scheduled next month.

### Case 3

A 15 year-old girl has complained for 8 months of right wrist pain and swelling. At presentation, she was 16 year-old. The right wrist was warm, effused and had decreased motion. She was diagnosed with oligoJIA. ANA and RF were negative. X-ray of the wrist was normal. The MRI revealed extensive carpal synovial proliferation, marked bone marrow edema, two bone cysts and bone erosion (see Fig. 1c). She started naproxen and physiotherapy, and the wrist joint was injected with the corticosteroid celestone.

Seven month after the diagnosis of oligoJIA, she was referred to our clinic. The right wrist was active. She required another two intra-articular celestone injections given 9 months apart. The MRI of the wrist done 1 month after the last injection showed improvement in synovial thicknening, resolution of bone marrow edema and reduction in one of the bone cysts. At last follow-up, she was asymptomatic on naproxen/esomeprazole magnesium.

### Case 4

A 9 year-old girl sustained a left supracondylar fracture 1 year prior to presentation to our clinic. Four weeks after the cast was removed, she continued to complain of elbow pain, and had decreased movements of the elbow. Despite intensive physiotherapy, the pain has continued and the movements of the elbow further decreased. At presentation, the left elbow was effused, tender, warm, and had significant flexure contracture. ANA was positive at 1:160, RF and anti-CCP antibodies were negative. PPD testing was negative. X-ray of the left elbow showed joint space narrowing and atrophy of the elbow’s articulation. CT of the left elbow demonstrated subchondral bone erosions, bone overgrowth and accelerated trochlear ossification (see Fig. 1d). The MRI revealed a small elbow effusion, marked synovial thickening, post-gadolinium enhancement of the radial head, trochlea, olecranon and coronoid bones, ballooning of the humeral epiphysis and multiple articular erosions. The child was started on naproxen, and the elbow joint was injected with triamcinolone hexacetonide. She also commenced intensive physiotherapy, including dynamic stretching and splinting. After 3 months, she was able to almost completely strengthen the elbow. A repeat MRI with gadolinium of left elbow is scheduled this month.

## Discussion

We report four children with oligoJIA that presented with monoarthritis of the hip, wrist and elbow. Three of them had not only cartilage loss, but also significant bone changes including ballooning of the adjacent bone, bone cysts and erosions.

Wrist and elbow involvement usually occur later in JIA. Only seven cases of wrist and three of elbow oligoJIA have been published to date [6–8]. The average age of these children was nine for wrist and 8 year-old respectively for elbow arthritis. Among these ten cases, eight (80%) were girls. The seven children with wrist oligoJIA (two boys and five girls) had either erosions on MRI or radiographic damage. The elbow arthritis in the three girls with oligoJIA was not associated with erosions by MRI.

In contrast, both our girls with wrist and elbow oligoJIA respectively had erosive arthritis at presentation. Moreover, both had symptoms for at least 8 months prior to diagnosis. This delay in diagnosis might have contributed to disease severity.

Hip involvement is commonly seen in enthesitis-related arthritis (ERA) and polyarticular JIA [9]. Isolated hip monoarthritis in children diagnosed with oligoJIA has not been reported yet. Moreover, HLA-B27 positivity has been associated with severe hip arthritis [10].

At presentation, both our girls with hip arthritis and positive HLA-B27 had already subchondral changes of the femoral head. In addition, the child that has been symptomatic for 8 months showed joint space loss. Although these girls met the ILAR classification criteria for oligoJIA [1], they did not classify as ERA. They had arthritis and a positive HLA-B27, but lacked a second criterium: history of SI joint tenderness, inflammatory lumbosacral pain or acute anterior uveitis, were girls, and had no first degree relatives with ankylosis spondylitis, ERA, sacroiliitis with IBD, Reiter’s syndrome or acute uveitis. However, under Amor’s [11] and the Assessment of SpondyloArthritis International Society (ASAIS)’s [12] criteria, their arthritis classifies as juvenile-onset spondylarthropathy (SpA). This might explain their unique presentation.

Limitations of the study. The small number of patients reported here precludes identification of predictor factors that may correlate with disease severity. The follow-up in two children was shorter than 6 months. Only 1 child was tested for anti-CCP antibodies. If the other three children have positive anti-CCP antibodies, this may explain, even partially, the aggressiveness of their arthritis [13]. However, given the severity of these rare cases, it is essential to share with the larger community of healthcare professionals and families their presentation and disease course.

## Conclusions

OligoJIA can present with hip, wrist and elbow arthritis. Girls with positive HLA-B27 presenting with isolated hip arthritis could meet the classification criteria for both oligoJIA and juvenile-onset SpA. This has implications for disease monitoring and management. Undiagnosed idiopathic monoarthritis can lead to joint and bone damage. Early referral to a pediatric rheumatologist, prompt diagnosis and treatment are essential for preventing irreversible joint loss, bones changes and growth impairment.

## Abbreviations

ANA: Antinuclear antibody
HLA-B27: Human leukocyte antigen B27
IAS: Intra-articular steroid injection
JIA: Juvenile idiopathic arthritis
MRI: Magnetic resonance imaging
OligoJIA: oligoarticular juvenile idiopathic arthritis
RF: Rheumatoid factor
